# Mesenchymal stem cells from sternum: the type of heart disease, ischemic or valvular, does not influence the cell culture establishment and growth kinetics

**DOI:** 10.1186/s12967-017-1262-0

**Published:** 2017-07-25

**Authors:** Lucinara Dadda Dias, Karina Rabello Casali, Carine Ghem, Melissa Kristocheck da Silva, Grasiele Sausen, Patrícia Bonini Palma, Dimas Tadeu Covas, Renato A. K. Kalil, Beatriz D. Schaan, Nance Beyer Nardi, Melissa Medeiros Markoski

**Affiliations:** 1 Programa de Pós-graduação em Ciências da Saúde-Cardiologia, Instituto de Cardiologia/Fundação Universitária de Cardiologia, Avenida Princesa Isabel, n° 370, 3° andar, Porto Alegre, RS CEP: 90620-001 Brazil; 2 Universidade Federal de São Paulo, São José dos Campos, SP Brazil; 3 Serviço de Patologia Clínica, Hospital de Clínicas de Porto Alegre, Porto Alegre, RS Brazil; 4 Laboratório de Citometria de Fluxo, Centro Regional de Hemoterapia do Hospital das Clínicas da Faculdade de Medicina de Ribeirão Preto/Universidade de São Paulo, São Paulo, SP Brazil; 5 Universidade Federal de Ciências da Saúde de Porto Alegre, Porto Alegre, RS Brazil; 6 Faculdade de Medicina, Universidade Federal do Rio Grande do Sul, Porto Alegre, RS Brazil; 7 Laboratório de Células-Tronco e Engenharia de Tecidos, Universidade Luterana do Brasil, Canoas, RS Brazil

**Keywords:** MSC establishment, Stem cells culture, Cell therapy, Valvular heart disease, Ischemic heart disease

## Abstract

**Background:**

In an attempt to increase the therapeutic potential for myocardial regeneration, there is a quest for new cell sources and types for cell therapy protocols. The pathophysiology of heart diseases may affect cellular characteristics and therapeutic results.

**Methods:**

To study the proliferative and differentiation potential of mesenchymal stem cells (MSC), isolated from bone marrow (BM) of sternum, we made a comparative analysis between samples of patients with ischemic (IHD) or non-ischemic valvular (VHD) heart diseases. We included patients with IHD (n = 42) or VHD (n = 20), with average age of 60 years and no differences in cardiovascular risk factors. BM samples were collected (16.4 ± 6 mL) and submitted to centrifugation with Ficoll-Paque, yielding 4.5 ± 1.5 × 10^7^ cells/mL.

**Results:**

Morphology, immunophenotype and differentiation ability had proven that the cultivated sternal BM cells had MSC features. The colony forming unit-fibroblast (CFU-F) frequency was similar between groups (p = 0.510), but VHD samples showed positive correlation to plated cells vs. CFU-F number (r = 0.499, p = 0.049). The MSC culture was established in 29% of collected samples, achieved passage 9, without significant difference in expansion kinetics between groups (p > 0.05). Dyslipidemia and the use of statins was associated with culture establishment for IHD patients (p = 0.049 and p = 0.006, respectively).

**Conclusions:**

Together, these results show that the sternum bone can be used as a source for MSC isolation, and that ischemic or valvular diseases do not influence the cellular yield, culture establishment or in vitro growth kinetics.

**Electronic supplementary material:**

The online version of this article (doi:10.1186/s12967-017-1262-0) contains supplementary material, which is available to authorized users.

## Background

In recent years, great attention has been given to the use of cell-based therapy in cardiology, with a number of clinical trials [1–3] that have been subjected to systematic reviews and meta-analyses [4–6]. Most of these studies have used bone marrow mononuclear cells (BMMC) obtained by iliac crest bone marrow (BM) aspiration [5]. The BM contains two types of adult stem cells: the hematopoietic stem cells give rise to blood cells [7], and the non-hematopoietic stem cells, or mesenchymal stem cells (MSC), contribute to the regeneration of mesenchymal tissues such as bone, cartilage and muscle [8]. In addition to their ability to differentiate into cellular components of the heart, such as endothelial cells and cardiomyocytes [9, 10], MSC are also able to release a great number of cytokines and chemokines, which can mediate the processes of tissue regeneration [8, 11]. For all these reasons, MSC have attracted increasing attention as a source of cells for cardiac regenerative therapy.

Since MSC are present in a low frequency in the BM (0.001–0.01%) [12], their use in clinical protocols requires ex vivo expansion and maintenance for 4–5 [13] or 6–8 weeks [14], depending on the pathology, so that a sufficient number of cells can be produced [15]. It is known that in aged individuals MSC have reduced frequency, capacity of expansion in culture [16, 17] and cellular fitness [18], factors that compromise the expansion of these cells in culture. Another problem relates to the limited capacity of expansion of BM cells after isolation and injection in patients who had had a myocardial infarction with consequent cardiomyocyte loss [19].

Considering that different heart diseases have different pathophysiological mechanisms, it is expected that cellular therapy for each of these diseases would have different results. This is the case of using cell therapy for heart failure determined by myocardial ischemia or by valve diseases, with BMMC or MSC [15, 20, 21]. The assessment of the ex vivo behavior of MSC isolated from elderly heart diseases patients with similar risk factors may provide valuable information for the choice of cell sources. The characterization of bone marrow-derived MSC is extremely important, since these patients represent of the main groups of interest for cell therapy and any loss in number or in cellular functionality can generate profound consequences for tissue regeneration processes.

The present study aims to quantify and analyze MSC isolated from sternal BM of patients with ischemic or non-ischemic valvular heart disease, evaluating the frequency of *colony forming unit*-*fibroblast* (CFU-F), the potential of establishing in vitro cultures and the kinetics of cultures until reaching senescence, as well as the differentiation potential. Clinical characteristics of patients, as well as the pharmacology in using, were also analyzed and correlated to the ability of establishment of cell cultures.

## Methods

### Patients

Patients with ischemic heart disease (IHD) or non-ischemic valvular heart diseases (VHD), between 50 and 75 years old, and referred for coronary artery bypass grafting or valve replacement surgery respectively, were included. Exclusion criteria were presence of hematologic diseases, previous heart complications and cancer diagnosis. The study was approved by the Research Ethics Committee of Instituto de Cardiologia (Process Number 4397/09), and was conducted in accordance with the Declaration of Helsinki. Written informed consent was obtained from all patients.

### Evaluation of clinical parameters

The clinical data were obtained from medical records, where we evaluated the age, the gender, the presence of systemic arterial hypertension (defined by blood pressure greater than 140/90 mmHg and by the use of antihypertensive medication), dyslipidemia (total cholesterol levels greater than 200 mg/dL, triglycerides grater than 150 and HDL-cholesterol grater than 40 for men and 50 for women, in addition to the use of lipid-lowering medication), diabetes mellitus (defined by glycemia exceeding 180 mg/dL and the use of oral hypoglycaemic or insulin), smoking (patients were considered smokers as declared *smoking* at the time of entering the study or who declared having stopped smoking until 10 years before entering the study). It was also considered the use of medications such as angiotensin-converting enzyme inhibitor, statins, antiplatelet drugs, diuretics, beta blockers and insulin.

### Isolation and cultivation of sternum MSC

The sternal BM was aspirated using a 10 mL syringe and 1.20 × 40 mm needles, with 1.5 mg EDTA/mL BM. BMMC were isolated by centrifugation over Ficoll-Paque Plus (GE Heathcare Life Sciences, Uppsala, Sweden). Cells from the mononuclear layer were washed, counted with trypan blue and resuspended in complete culture medium, composed of low-glucose Dulbecco’s modified Eagle’s medium (DMEM, Gibco-Carlsbad, SP, Brazil) with 15% fetal bovine serum (Cultilab, SP, Brazil), 100 U/mL penicillin and 100 mg/mL streptomycin (Cultilab). Cells were plated in duplicate samples in 12-well culture plates, at 2.8 × 10^6^ BMMC/cm^2^ and incubated at 37 °C in a humidified, 5% CO_2_ incubator for 72 h, when non-adherent cells were removed by changing the medium. The medium was changed twice weekly. For expansion of cultures the cells were passaged (split) when they reached 80–85% of area confluence. For this, the medium was removed and adherent cells were washed twice with phosphate-buffered saline (PBS, pH 7.4) and incubated with 0.05% Trypsin–EDTA (Gibco) for about 5 min at 37 °C. Cultures were considered successful when reaching the passage 3 (P3). Plastic ware was from Becton–Dickinson (BD Biosciences, San Jose, CA, USA).

### Proliferation kinetics

MSC were analyzed for proliferation capacity in two stages. In the first one, BMMC were initially plated in duplicate samples in 12-well culture plates, at 2.8 × 10^6^ cells/cm^2^ and passaged at 80–85% confluence. From P1–P3, cells were plated at different densities (10, 18 and 75 × 10^3^ cells/cm^2^, respectively). From passage 4 on, a protocol adapted from Stolzing et al. [18] was used. Briefly, MSC were plated in triplicate samples in 6-well culture plates at 10^4^ cells/cm^2^ and passaged at 80–85% confluence at the same density. Cells were maintained in culture and counted at every passage, until proliferation stopped.

The population doubling (PD), cumulative population doubling (CPD) and cumulative days were determined as previously described [22]. The population doubling time (PDT) was calculated at P3 and P9 as *t*/n, where *t* is the duration of culture in days and n is the number of PD.

### Immunophenotyping

Cells were used in P3, centrifuged, and incubated for 30 min at room temperature in the dark with PE-conjugated (anti-CD49e, CD166, CD14, CD146, HLA-ABC, CD105 and CD73), FITC-conjugated (anti-CD31, CD45, CD51/CD61, CD44, CD29, HLA-DR, CD90 and CD106), PerCP-conjugated anti-CD34, APC-conjugated anti-CD13 antibodies and respective isotype controls (BD Biosciences). The cells were analyzed using a FACS Calibur cytometer (Becton–Dickinson, San Diego, CA, USA) with the CellQuest Pro software. At least 10,000 events were collected. Forward scatter (FSC) and side scatter (SSC) were used for gating on staining cells and excluding cell debris.

### MSC differentiation

MSC cultures (P3) at 80% confluence were kept for 21 days in osteogenic- or adipogenic-inducing media [23, 24]. Controls were maintained in standard medium for the same period. Experiments were performed in triplicates. Differentiation was analyzed with Alizarin Red S or Oil Red *O* (Sigma-Aldrich, St Louis, MO), respectively, according to Kretlow et al. [24]. In each well, five randomly selected fields were photographed with an AxioCam camera (Carl Zeiss, Hallbergmoos, Germany). Stained areas were quantified using the Image-Pro Plus software and expressed as percentage of the total analyzed area.

### Colony forming unit-fibroblast assay

The CFU-F assay was performed as previously described [18, 25]. BMMC were resuspended in complete culture medium and plated in duplicate samples in 6-well culture plates, at 5 × 10^5^, 10^6^ and 10^7^ BMMC/well. Medium was changed on days 3 and 8 of culture. On day 14th, cultures were fixed and stained with May-Grünwald-Giemsa and colonies (groups of >30 fibroblastoid cells) were counted. The CFU-F frequency was calculated based on the respective input cell number as CFU-F per 1 × 10^6^ BMMC.

### Statistical analyses

The data were tested for normality using a Kolmogorov–Smirnov test. The results are expressed as mean ± standard deviation (SD) or median and interquartile intervals. Groups were compared using parametric tests (Student’s *t* test) for CPD, cumulative days, and CFU-F or non-parametric tests (Mann–Whitney test) for analysis of PDT and MSC differentiation. The Chi square test was used to compare the rate of success in establishing MSC cultures, and the Fisher’s exact test for comparing characteristics between groups. The level of significance was set at 5% for all tests. Statistical analyses were performed with the SPSS software package, version 19.0, and Graphpad Prism 5.

## Results

### Characterization of the sample

A total of 62 patients were included in the study (IHD, n = 42; VHD, n = 20). As presented in Table 1, most characteristics were similar between groups. The use of diuretics was higher in the VHD group, male sex frequency, statins use, use of antiplatelets and beta-blockers was higher in IHD patients, as expected.

**Table 1:** Clinical characteristics of patients with ischemic (IHD) or non-ischemic valvular heart disease (VHD)

Variables	IHD (n = 42)	VHD (n = 20)	p
Age (years)	59.30 ± 6.61	62.35 ± 8.82	0.135
Male	37 (88.1)	13 (65.0)	0.043
Hypertension	34 (81.0)	14 (70.0)	0.349
Diabetes mellitus	13 (31.0)	3 (15.0)	0.226
Smoking	30 (71.4)	10 (50.0)	0.155
Dyslipidemia	6 (14.3)	3 (15.0)	1.000
Medications in use			
Angiotensin-converting enzyme inhibitor	26 (61.9)	13 (65.0)	1.000
Statins	28 (66.7)	7 (36.8)	0.049
Antiplatelet drugs	30 (71.4)	7 (35.0)	0.012
Diuretics	6 (14.3)	12 (60.0)	0.001
Insulin	1 (2.4)	1 (5.0)	0.545
Beta-blockers	30 (71.4)	6 (30.0)	0.002

Age described as mean ± standard deviation; other variables as n and percentage (%)

*IHD* ischemic heart disease, *VHD* non-ischemic valvular heart disease

Statistical analysis by Fisher’s exact test (α = 5%)

### MSC from sternum of IDH and VHD patients can be established and cultivated in vitro

**Fig. 1:**
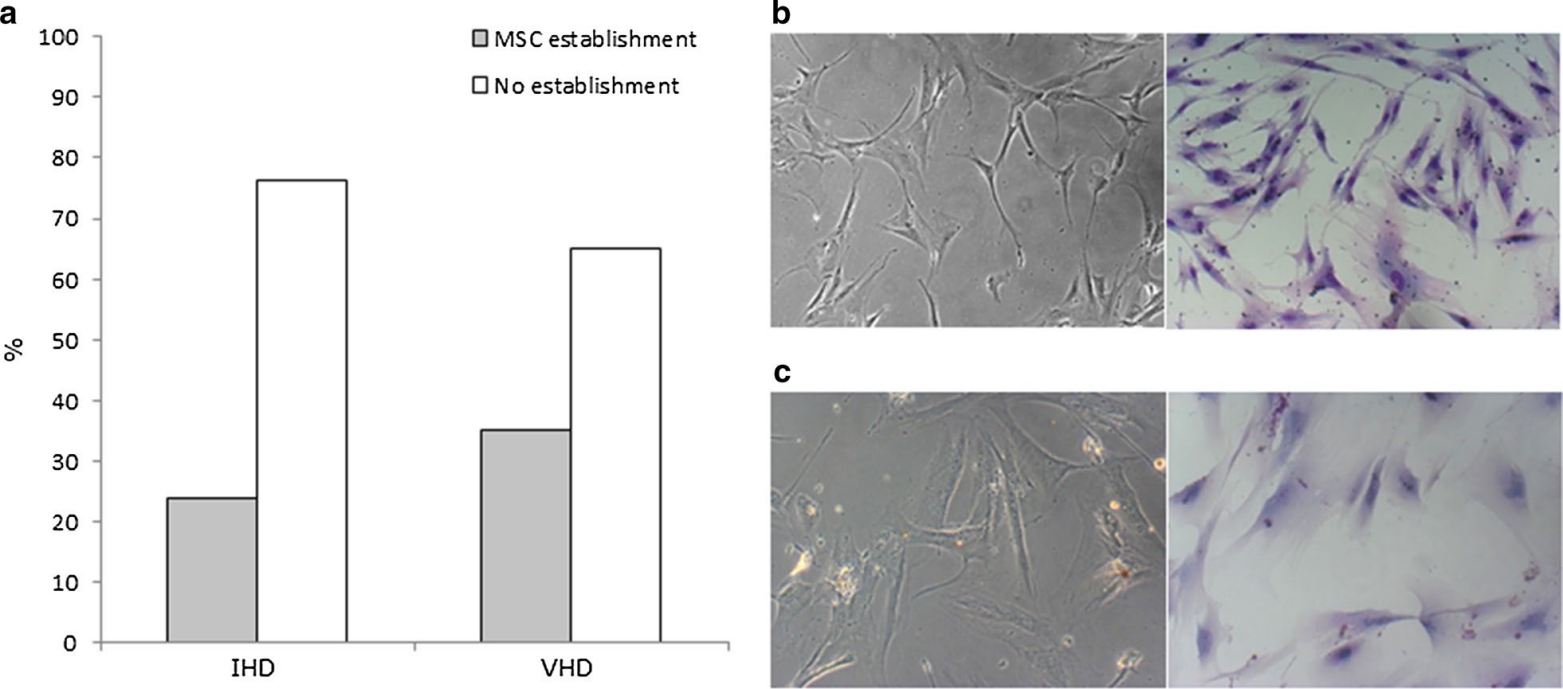
Quantification analysis of sternum MSC establishment and cell morphology. **a** Percentages of samples with or without establishment of cell cultures (overcoming P3) for ischemic (IHD) and non-ischemic valvular heart disease (VHD) (p = 0.189). **b** Young (P1) and **c** older (P9) cultures, under phase contrast microscopy (*left*) and stained with May-Grünwald-Giemsa (*right*). Original magnifications ×100

The mean volume of sternal BM collected was 16.4 ± 6 mL, which yielded in average 4.5 ± 1.5 × 10^7^ cells/mL of tissue. We considered a successful establishment when cultures reached the P3. However, between the 42 IHD samples, 6 reached only P1 and other 3 samples stop proliferation before P2; and, in 20 isolated samples of VHD patients, 2 stopped proliferation before P2, being in culture for up to 40 days. Thus, of the 62 samples analyzed, the successful establishment of cultures characterized as MSC was 29% (n = 18). The percentage of MSC established cultures did not differ (p = 0.189) between the groups IHD and VHD (23.8%, n = 10, and 40%, n = 8, respectively) (Fig. 1a). Considering the influence of the clinical parameters in the MSC establishment, we found that the presence of dyslipidemia, as well as patients who used statins, both in the IHD group, were features that were significantly associated with the presence of in vitro cell cultivation (p = 0.049 and p = 0.006, respectively). No other parameter (reported in Table 1) showed association with the presence or the absence of culture establishment of MSC. The same was observed when patients of IHD and VHD groups were considered separately or grouped (p > 0.05). Young cultures (P1 to P3) showed a fibroblast-like morphology, with a well-delimited nucleus and with regular borders (Fig. 1b). In older cultures (≥P3), the fibroblastic morphology was modified, and larger cytoplasms and irregular borders as well as intracellular debris were observed (Fig. 1c).

### Cultured cells from sternum of IHD and VHD patients have characteristic MSC immunophenotype and ability to in vitro differentiation

**Fig. 2:**
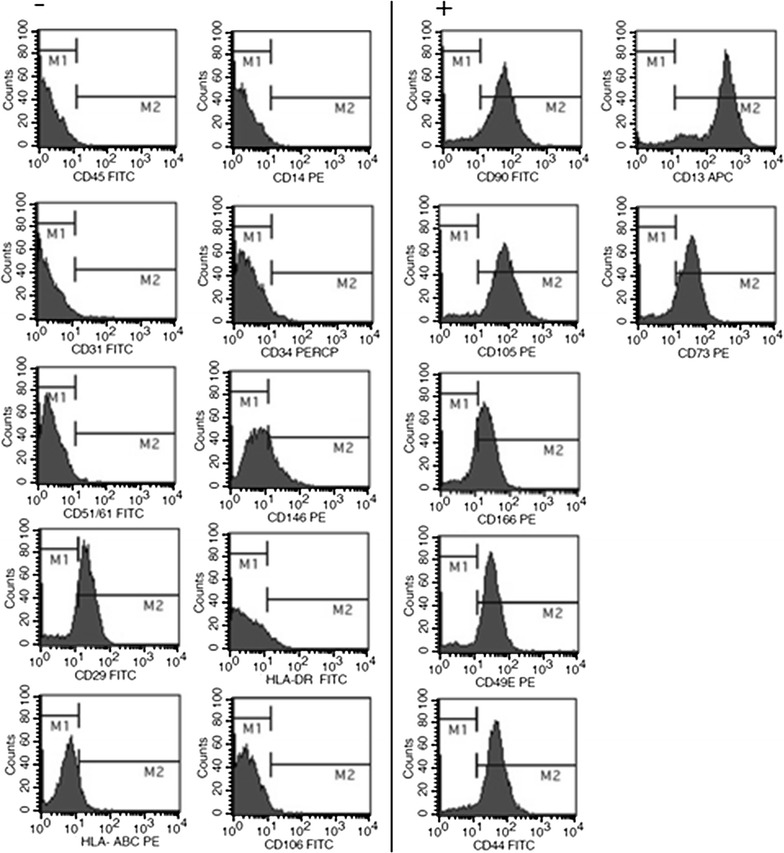
Representative immunophenotype profile of MSC cultures isolated from sternum of ischemic (IHD) and non-ischemic valvular heart disease (VHD). Positive (*right*) and negative (*left*) staining for MSC, hematopoietic and endothelial cell populations analyzed by flow cytometry

**Fig. 3:**
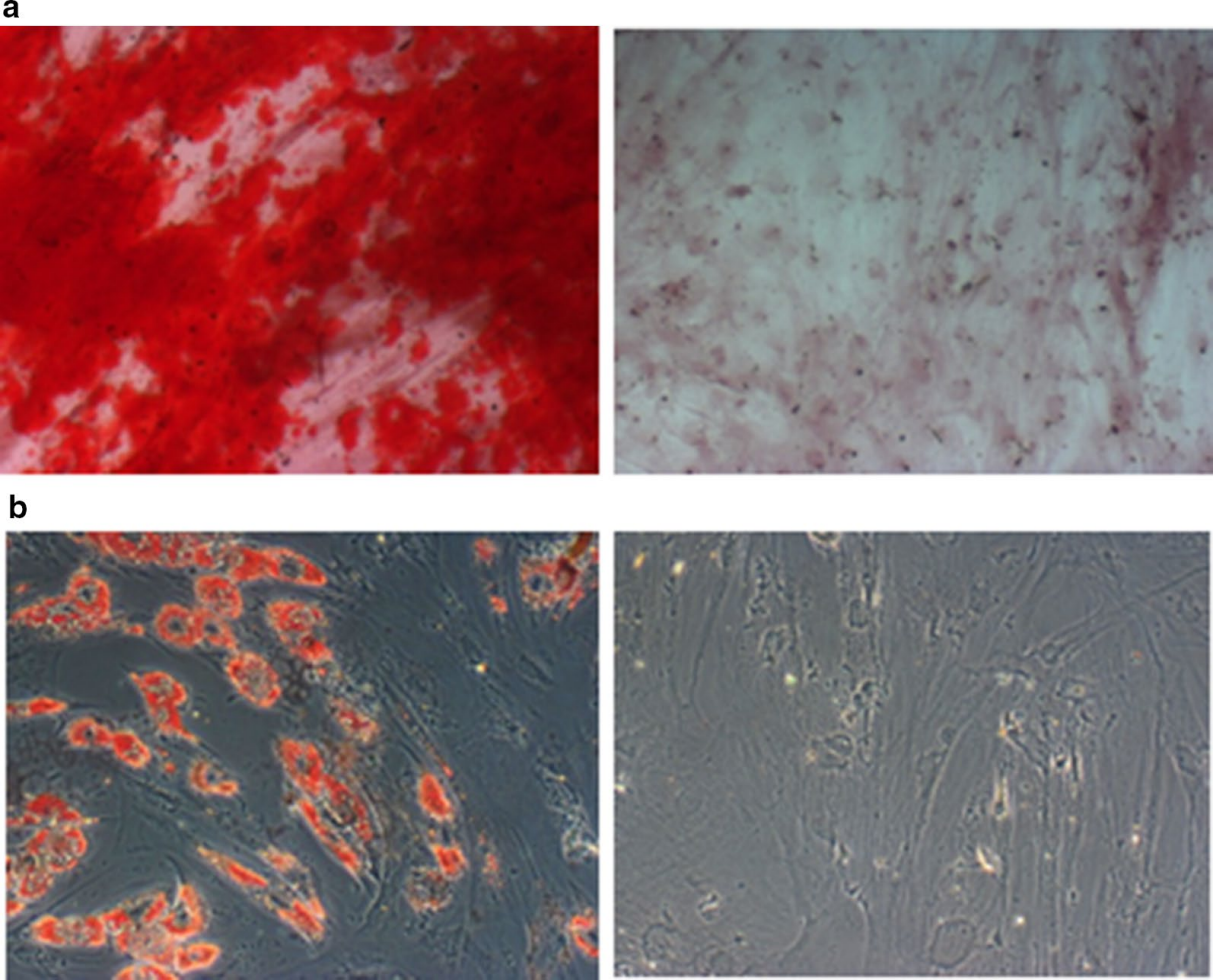
Differentiation ability of MSC cultures from sternum of ischemic (IHD) and non-ischemic valvular heart disease (VHD) patients. Representative micrography of positive (induced, *right*) and negative (control, *left*) staining for **a** Alizarin Red S (osteogenic marker) and for **b** Oil Red *O* (adipogenic marker). Original magnifications ×100

MSC cultures isolated from IHD and VHD patients showed a characteristic surface profile, with low or no expression (<2% of events) of hematopoietic (CD34, CD14, CD45) and endothelial (CD51/61, CD31, CD146) markers and HLA-ABC, as well as of HLA-DR and CD106, and presence of CD73, CD105, CD166, CD90, CD13, CD29, CD49e, CD44 (Fig. 2). The analysis of cell size and granularity (FSC and SSC, respectively) and counts for antibody-isotype controls may be seen in the Additional file 1. Further, in samples from both groups, the osteogenic and adipogenic differentiations were observed in 60 and 75%, respectively, of MSC cultures treated with inducing media comparing to controls (Fig. 3). Besides, there was no difference between the adipogenic (p = 0.461) or osteogenic (p = 0.635) differentiation rates for samples isolated from patients with IHD and VHD.

### The type of heart disease does not affect proliferative capacity of MSC isolated from sternum

#### Quantification of CFU-F frequency

**Fig. 4:**
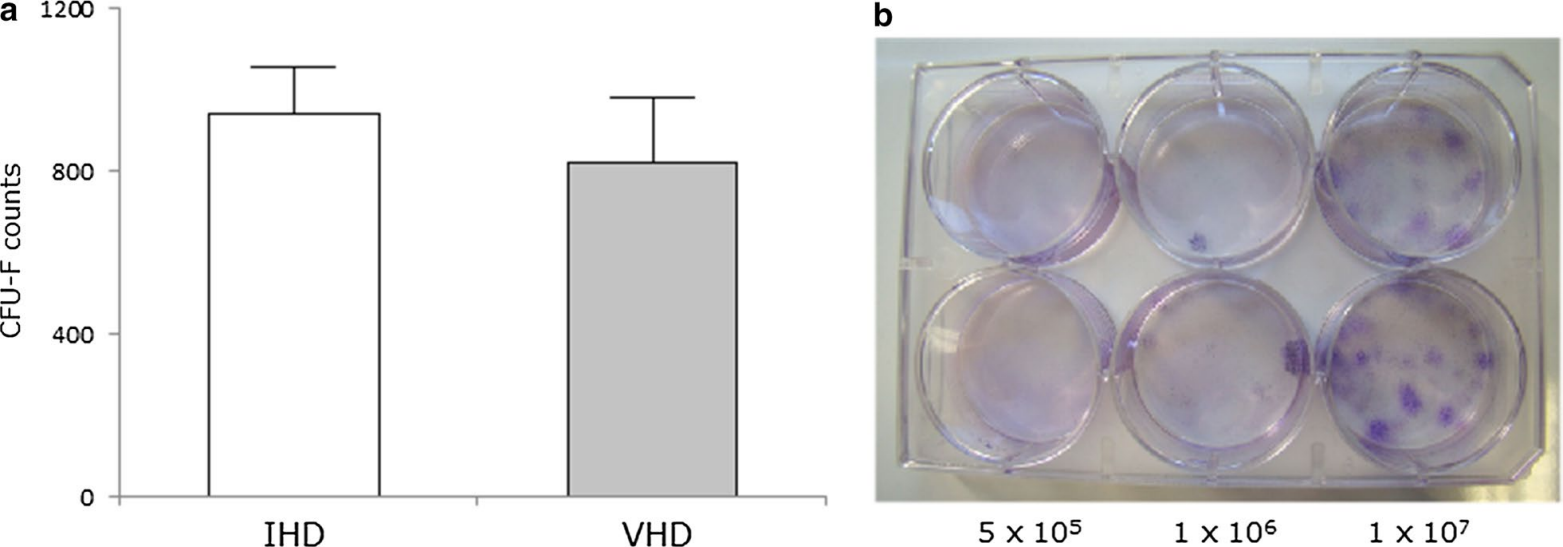
CFU-F counts from sternal BM of ischemic (IHD) and non-ischemic valvular heart disease (VHD) patients. The BMMC separated from sternal BM were seeded in values varying between 5 × 10^5^ and 1 × 10^7^ cells. **a** *Bars* show the average number of colony forming unit-fibroblast (CFU-F) counted after 14 days and related to 1 × 10^6^ BMMC seeded for each disease. **b** Representative image of CFU-F from IHD (*upper* wells) and VHD (*bottom* wells) samples in different concentrations of seeding

Figure 4 shows the average CFU-F count obtained from BMMC samples isolated from sternum of patients with IHD or VHD. The CFU-F frequencies were similar to both cardiopathies, being 1/1.1 × 10^6^ and 1/1.2 × 10^6^, respectively (p = 0.510). There was a positive correlation (r = 0.499; p = 0.049) between the number of plated cells and the number of CFU-F in the VHD group, however, the same did not occur for the IHD group (r = 0.188; p = 0.302).

#### Proliferation and expansion kinetics

**Fig. 5:**
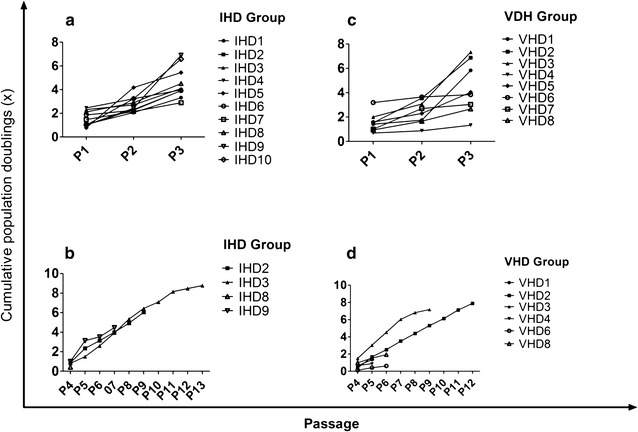
Proliferation kinetics of MSC isolated from sternum of ischemic (IHD) and non-ischemic valvular heart disease (VHD) patients. Cumulative population doubling (CPD) of individual MSC cultures derived from IHD (**a**, **b**) or VHD (**c**, **d**) samples. **a**, **c** From P1 to P3. **b**, **d** From P4

**Fig. 6:**
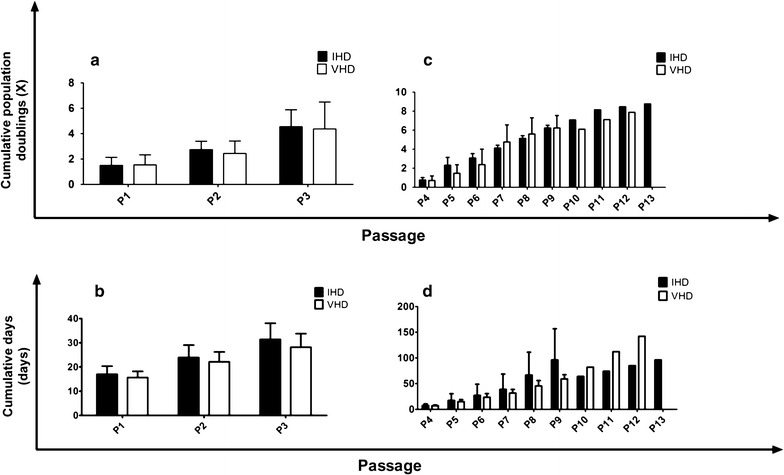
In vitro expansion kinetics of MSC isolated from sternum of ischemic (IHD) and non-ischemic valvular heart disease (VHD). **a**, **c** Average cumulative population doubling (CPD) and cumulative days in each passage of IHD and VHD samples. **a**, **b** From P1 to P3. **c**, **d** From P4. Sample size (n) is shown in Fig. 5

From P1 to P3, the expansion rates of individual MSC cultures derived from IHD and VHD samples showed a large variation, with eightfold duplication of the initial population in some of the cultures and only one in others (Fig. 5a–c). From passage 4 on, however, cultures showed a low proliferation capacity or stopped proliferation (Fig. 5b–d). Four of the 10 established cultures from IHD samples, were maintained after P4, and only three were maintained after P7 (Fig. 5a, b). Six of the 8 MSC cultures established from VHD samples were maintained after P4 and only two after P6 (Fig. 5c, d). No differences were observed between the two groups of cultures regarding the expansion rate (CPD, Fig. 6a–c) in each passage (P1, p = 0.905; P2, p = 0.447; P3, p = 0.827; P4, p = 0.839; P5, p = 0.221; P6, p = 0.514; P7, p = 0.703; P8, p = 0.740; P9, p = 1.00). No differences were observed between the two groups in the number of days to reach 80–85% confluence (cumulative days, Fig. 6b–d) in each passage (P1, p = 0.351; P2, p = 0.439; P3, p = 0.283; P4, p = 0.789; P5, p = 0.757; P6, p = 0.771; P7, p = 0.881; P8, p = 0.583; P9, p = 0.484), or in the PDT measured at P3 (IHD, 7.7 days to 5.5–8.5 days—vs. VHD, 6.5 days to 4.5–8.8 days; p = 0.696) or P9 (IHD, 11.1 days to 5.2–15.8 days—vs. VHD, 7.4 days to 3.7–16.5 days; p = 0.730).

## Discussion

The present study described for the first time the frequency and the functionality of MSC isolated from the sternal bone marrow of two groups of patients with distinct heart diseases. We showed that cultures showed characteristics compatible to those established by the International Society for Cellular Therapy, including proliferation as plastic-adherent cells, immunophenotype (expression of CD105, CD73 and CD90 and low or no expression of CD45, CD14, CD34 and HLA-DR) and the capacity differentiation into mesodermal cell lineages [26]. These results show that the sternum of patients with IHD or VHD can be a source for collecting both BMMC and MSC, and that the type of heart disease does not influence the establishment of cultures. Since it is easier to collect sternal than femoral bone marrow in patients undergoing median sternotomy, this alternative source can be considered in cases of autologous cell therapy.

The proliferative capacity of MSC used in our study was confirmed in the first weeks and was maintained throughout the period of cultivation. Cultures were established from 30% of the samples, which could be explained by the fact that these are elderly patients, with lower bioavailability of red bone marrow and augmented cellular aging, which causes DNA damage, apoptosis and a reduction of the pool of undifferentiated cells and, consequently, proliferative potential [27, 28]. However, no association was observed between age and establishment of culture (p = 0.465). On the other hand, the establishment of MSC cultures was positively associated with presence of dyslipidemia in IHD patients and the use of statins. It is known that statins are able to modulate endothelial progenitor cells, another example of adult stem cell, increasing their in vitro proliferative capacity [29]. In contrast, the presence of other factors inherent to heart diseases does not seem to affect the ability of establishment of MSC cultures.

The low frequency of CFU-F (1/1 × 10^6^ MSC/BMMC) observed in the present study can also be related to low proliferative potential of cells isolated from the sternal compartment. The initial expansion of cultures is a result of CFU-F present in the sample (single cells that continue to divide to form colonies of cloned cells) [7], so that a lower number of colonies suggests a lower success in the establishment of cultures, besides changes in the functional capacity of MSC [18]. Age has been already shown to be closely related to the number of CFU-F, as reported by several studies [16, 18, 30, 31]. Baxter et al. [30], for example, reported a great difference in the number of CFU-F in BM samples from healthy donors of old age (59–75 years) and pediatric donors (0–18 years) (3.2 ± 1.7/10^6^ vs. 29.0 ± 4.7/10^6^ BMMC, respectively). Our study showed that the frequency of CFU-F obtained from the sternal BM of heart disease patients was approximately two times smaller. These results suggest that not only increasing age, but also the presence of specific factors associated with cardiovascular disease—such as molecules and drugs used that may affect common pathways to the proliferation and homing process—could decrease the regenerative capacity of endogenous BM stem cells [27]. Ito and Suda [32] suggested that metabolic requirements of the BM, which direct adult stem cells to anaerobic glycolysis (keeping the cells undifferentiated) or oxidative metabolism (which activates differentiation), can control the process of exhaustion of the stem cell compartment. Thus, the stem cell compartment is directly affected by to the life history of the cardiac patient, related to maintenance of inflammatory states that disrupt homeostasis and accelerates regenerative processes.

Cultures established from sternal BM of the patients were also analyzed for morphology. During the initial period (P0–P3), the cells presented a fibroblastoid morphology, but in later passages, modifications suggestive of senescence were observed. These changes included loss of fibroblastoid aspect, presence of irregular borders, wide cytoplasm and presence of intracellular debris. These differences were also reported for human MSC cultures isolated from the BM of healthy individuals [31] and for cultures of trabecular osteoblasts [33], suggesting that they are imposed by the two-dimensional in vitro cultivation condition. Cultures older than P3–P4 were more difficult to expand and/or lost the ability to proliferate, which is important since, even aiming at the use of MSC (which are considered the adult stem cells with greater therapeutic potential), clinical protocols for cardiovascular diseases require large cell numbers [34]. This decrease in proliferative potential of MSC was also reported by Choumerianou et al. [31], who observed that MSC isolated from children (4 ± 1 years old) were more easily expanded until, at least, P6 than cells from healthy adults (54 ± 5 years old), which were more difficult to expand after P4. The authors have also observed a relationship of these findings with shortening of telomeres presented by MSC from adults. Telomeres are known to play an important role in the molecular aging process of cells [35].

Few studies have compared cells isolated from different sites such as sternal and iliac crest. Adams et al. [36] showed that MSCs isolated from equine sternum and ilium had similar characteristics, without difference between growth rates. In sheep, the sternum was considered a good source of bone marrow MSC, with cell division cycle and proliferative potential similar to the cells from iliac bones, showing that a sample from one site is representative of the whole [37]. Human stromal stem cell populations reside in different tissues and the human MSC isolated from iliac crest, sternum and vertebrae bone marrow have also shown similar immunophenotype but different growth and differentiation abilities, suggesting that they may not represent equivalent cell sources for therapeutic applications [38].

The limitations of our study are consistent to those involving collections of biological samples from patients (different sample collectors, different locations of the sternum for bone marrow aspiration, different volumes collected, etc.). Later, the influence of the pathophysiological condition of the patient (presence of disease and associated factors) lead to low proliferative capacity of isolated cells, mainly in a two-dimensional system for in vitro growth, which in our case, represented approximately 30% of success in establishment. Anyway, in order to assist the medical conduct intending to use protocols based on isolated cells of the bone marrow, our group is also investigating the mechanisms involved with the homing of stem cells, mainly focused on the modulation of the chemokine stromal-derived factor-1 (SDF-1) and its receptor, CXCR4, beyond the inflammatory profile of patients. This data may identify if the cells are capable of being stimulated to cell migration and settlement in cardiac tissue differentially in ischemic and valvular cardiopathies through the expression of these molecules.

## Conclusions

We showed that it is possible to isolate and cultivate MSC from small volumes of sternal bone marrow of cardiac patients. Valvular and ischemic heart diseases, even with variable pathological and physiological aspects, do not have different influences on the proliferative capacity or differentiation potential of the isolated MSC, so that the autologous cell therapy protocols with these cells can be designed for both diseases. However, the processes of expansion and exhaustion of the BM stem cell population are probably influenced/modulated by risk factors such as diabetes, hypertension, smoking, obesity and dyslipidemia, in addition to pharmacological therapy. These factors must be considered, as they may contribute to the failure of the proliferation of MSC cultures, necessary for autologous cell therapy. However, these cells can be safely isolated, and used in in vitro experiments, such as tests for drugs, besides transcriptome and proteome analyses according to the different stages of the disease and patient condition. Additionally, MSC are known for their high potential for regeneration and secretion of paracrine factors [39]. In this context, the discovery of mechanisms that can modulate and increase the therapeutic capacity of the MSC is a constant objective in this area of research, which aims at better therapeutic strategies for the therapy of cardiovascular diseases.

## Additional file

**Additional file 1.** Flow cytometry analysis of size and granularity (FFS x SSC) and staining of antibody-isotypes controls.

## Abbreviations

BM: bone marrow
BMMC: bone marrow mononuclear cells
CFU-F: colony forming unit-fibroblast
CPD: cumulative population doubling
IHD: ischemic heart disease
MSC: mesenchymal stem cell
PDT: population doubling time
VHD: non-ischemic valvular heart disease
