# Supplementary figures and images for: Mesenchymal stem cells from sternum: the type of heart disease, ischemic or valvular, does not influence the cell culture establishment and growth kinetics
Source: J Transl Med. 2017 Jul 25;15:161. doi: 10.1186/s12967-017-1262-0 (PMC5526254; doi:10.1186/s12967-017-1262-0)

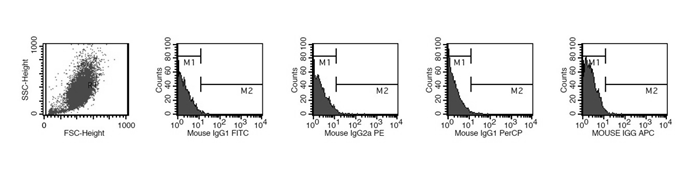

Supplement: Supplementary file 1 — Additional file 1. Flow cytometry analysis of size and granularity (FFS x SSC) and staining of antibody-isotypes controls. [file 12967_2017_1262_MOESM1_ESM.tif]
